# Secoisolariciresinol diglucoside alleviates inflammatory injury in osteoarthritis chondrocytes through ERBB2-associated JAK2/STAT3 signaling: network pharmacology and experimental validation

**DOI:** 10.3389/fimmu.2026.1891212

**Published:** 2026-08-26

**Authors:** Haibei Hu, Gaoshan Li, Yangxu Wang, Suyu Xie, Feng Cao, Huanyu Li, Xubin Gao

**Affiliations:** 1 Department of Orthopaedics, The First Affiliated Hospital of Bengbu Medical University, Bengbu Medical University, Anhui Key Laboratory of Tissue Transplantation, Bengbu, China; 2 Department of Orthopaedics, 968 Hospital of Joint Service Support Force of Chinese People’s Liberation Army, Jinzhou, China

**Keywords:** chondrocyte, ERBB2, JAK/STAT signaling, osteoarthritis, secoisolariciresinol diglucoside

## Abstract

**Background:**

Cartilage injury is a hallmark of osteoarthritis (OA). erb-B2 receptor tyrosine kinase 2 (ERBB2) has been reported to be involved in mediating the therapeutic effects of polyphenols in orthopedic diseases. This study aimed to investigate the protective effects and underlying mechanisms of action of ERBB2 in OA chondrocytes.

**Methods:**

Bioinformatics analysis and molecular docking were used to predict the binding relationship between ERBB2 and the polyphenolic compound, secoisolariciresinol diglucoside (SDG). ATDC5 cells were induced with insulin-transferrin-selenium (ITS), followed by lipopolysaccharide (LPS) exposure to mimic OA inflammation, and treated with SDG. Cell viability, collagen II expression, apoptosis, interleukin 6 (IL-6)/Tumor Necrosis Factor-α (TNF-α) secretion, and malondialdehyde (MDA)/superoxide dismutase (SOD) levels were evaluated using cell counting kit-8 (CCK-8), immunofluorescence, flow cytometry, enzyme-linked immunosorbent assay (ELISA), and commercial kits. Matrix metalloproteinase 13 (MMP13) and ERBB2 mRNA levels were assessed by quantitative real-time polymerase chain reaction (qRT-PCR). Extracellular matrix integrity was assessed by toluidine blue staining. The predicted SDG-ERBB2 interaction was validated using a cellular thermal shift assay (CETSA). Western blotting was performed to assess the expression of ERBB2 and JAK2/STAT3. Rescue experiments with ERBB2 overexpression or colivelin (a JAK/STAT activator) treatment confirmed the involvement of this pathway.

**Results:**

SDG dose-dependently reversed the LPS-induced reduction in cell viability and collagen II expression, while suppressing apoptosis, IL-6/TNF-α secretion, and MDA levels, and increasing SOD activity. SDG significantly reduced MMP13 mRNA levels and enhanced extracellular matrix proteoglycan deposition, indicating the preservation of matrix integrity. ERBB2 has been identified as a candidate target for SDG. SDG inhibits ERBB2 protein expression and reduces JAK2 and STAT3 phosphorylation ERBB2 overexpression and colivelin treatment abrogated the protective effects of SDG on cell viability, collagen II expression, apoptosis, inflammation, and oxidative stress, as well as its suppression of MMP13 and preservation of matrix integrity.

**Conclusion:**

SDG protects chondrocytes against LPS-induced inflammatory injury by targeting ERBB2 and suppressing the downstream JAK2/STAT3 signaling pathway. These findings provide mechanistic evidence supporting the chondroprotective potential of SDG under inflammatory conditions and suggest that ERBB2/JAK2/STAT3 signaling may represent a potential therapeutic target in OA.

## Introduction

Osteoarthritis (OA) is a chronic degenerative joint disease characterized by gradual degradation of the articular cartilage (1). As the most common type of arthritis, OA is one of the main causes of dysfunction and decline in patients’ quality of life (1). At present, drug therapy for OA (such as nonsteroidal anti-inflammatory drugs) mainly relieves symptoms, but it cannot halt disease progression and is often associated with significant adverse reactions, highlighting the urgent need for safer and more effective alternative strategies (2).

In this context, nutritional health products containing bioactive compounds have become a promising research topic (3). Among them, polyphenols, as secondary metabolites of plants, have remarkable anti-inflammatory and anti-oxidation abilities and have been widely studied in preclinical OA models and clinical environments (4). As a subclass of polyphenols, lignans have been proved to resist inflammation, oxidative stress and bone degeneration (5, 6). Notably, secoisolariciresinol diglucoside (SDG), a purified lignan monomer compound derived from plants such as flaxseed, has anti-inflammatory and anti-apoptosis activities (7). Although SDG has been reported that SDG can alleviate OA (8), its specific molecular target remains unclear.

The pathophysiological process of OA involves complex remodeling of the extracellular matrix (ECM) of the articular cartilage (9). Recent studies have emphasized the role of focal adhesion (FA) pathway in bone formation and the maintenance of cartilage integrity (10). The FA protein is an integrin-related adaptor that transmits mechanical and biochemical signals between the ECM and cytoskeleton and plays an important role in the proliferation, differentiation, and survival of chondrocytes (10). Among FA-associated molecules, Erb-B2 Receptor Tyrosine Kinase 2 (ERBB2), a member of the epidermal growth factor receptor (EGFR) family (11, 12), has recently attracted attention in OA research, and the decreased expression level of ERBB2 is related to decreased expression of catabolic-related genes and decreased inflammatory response (13). Therefore, inhibition of ERBB2 may have therapeutic potential in OA.

The Janus kinase/signal transducer and activator of transcription (JAK/STAT) pathway is an evolutionarily conserved signal transduction mechanism that regulates apoptosis and is closely related to the pathogenesis of OA (14). Importantly, ERBB2 activates JAK/STAT signaling (15). In addition, there is growing evidence that interleukin-6 (IL-6), an important inflammatory mediator, connects upstream receptor activation with the JAK2/STAT3 signaling pathway. IL-6 can induce JAK2 activation by binding to IL-6 receptor (IL-6R) and gp130, thereby promoting STAT3 phosphorylation and transcription of inflammation-related genes (16, 17). Previous studies have shown that the activation of ERBB2 can enhance the production of IL-6 through the downstream inflammatory signaling cascade, thus forming a positive regulatory loop to maintain the activation of JAK2/STAT3 (18). In OA, elevated IL-6 levels promote chondrocyte catabolism by inducing matrix degradation, inflammatory responses, and cartilage destruction (19). Therefore, IL-6 may be a key intermediate between ERBB2 and JAK2/STAT3 pathway activation and plays an important role in the progression of OA. Based on these findings, it is suggested that the activation of JAK/STAT, which is over-driven by ERBB2, is involved in chondrocyte damage in OA, and targeting this signaling axis may produce therapeutic benefits.

Therefore, the aim of this study was to investigate whether SDG protects chondrocytes against inflammatory injury and to explore the potential involvement of ERBB2-associated JAK2/STAT3 signaling in this process. These findings provide mechanistic insights into the chondroprotective properties of SDG and support future studies evaluating its potential relevance in OA-related conditions.

## Materials and methods

### Cell culture

ATDC5 cells were purchased from the American Type Culture Collection (ATCC). These cells were cultured in Dulbecco’s Modified Eagle’s medium (DMEM, Thermo Fisher, USA) supplemented with 10% fetal bovine serum (FBS, Thermo Fisher, USA). The culture conditions is the standard condition: 37 °C, moist atmosphere with 5% CO_2_. To induce chondrogenic differentiation, cells were pretreated with 1% insulin-transferrin-selenium (ITS; China Biyuntian Company) for 14 days (20) before all subsequent cell-based experiments began. This ensured the induction of the mature chondrocyte phenotype.

### Cell treatment

After chondrogenic induction, ATDC5 cells were subjected to OA modeling by incubation with 5 μg/mL lipopolysaccharide (LPS; Med Chem Express, China) for 24 h to mimic the inflammatory microenvironment of OA (20). In line with previous studies (8), chondrocytes were treated with SDG (Med Chem Express, China) for 24 h at doses of 0, 5, 10, 25, and 50 μM. For rescue experiments, cells were pretreated with 0.5 μM colivelin (a specific activator of the JAK/STAT signaling pathway; Med Chem Express, China) for 48 h (21) before SDG and LPS treatment.

ERBB2 overexpression plasmid was purchased from Gene Pharma (Shanghai, China). For transfection, 6-well plates were seeded with cells, and the ERBB2 overexpression plasmid was introduced using Lipo8000 (Beyotime, China), following the supplier’s protocol. Subsequently, the transfected cells were treated with SDG and LPS as described above.

### Cell viability assay

The Cell Counting Kit-8 assay (CCK-8, MedChemExpress, China) was used to assess cell viability. Cells were plated in 96-well plates at 3000 cells/well and exposed to the interventions described above. After the exposure period, each well received 10 μL of CCK-8 solution, followed by a 2-hour incubation at 37 °C. Absorbance was measured at 450 nm using a microplate reader (BioTek, USA).

### Immunofluorescence staining

Following previously described treatments, cells were seeded onto coverslips placed in 24-well plates. For fixation, 4% paraformaldehyde (PFA; Beyotime, China) was applied for 15 min at ambient temperature. Thereafter, membrane permeabilization was achieved with 0.1% Triton X-100 (Coolaber, China) over a 10-minute period, followed by a blocking step using 5% bovine serum albumin (BSA, Elabscience, China) for 1 h. The specimens were then incubated overnight at 4 °C with a primary antibody specific for Collagen II (Abcam, Cambridge, UK). After rinsing with PBS, FITC-conjugated secondary antibody (Abcam) was added for 1 h under dark conditions at room temperature. Nuclear staining was performed with DAPI (; Coolaber, China) for 5 min. Finally, the coverslips were mounted with an anti-fade mounting medium (Coolaber, China) and fluorescence images were captured using a microscope (Olympus, Japan).

### Flow cytometry

The apoptotic rate of chondrocytes was measured within 1 h using a flow cytometer (Accuri C6 Plus, BD, USA). For detection, the cells were collected after treatment and suspended in binding buffer at a density of 1×10^6^ cells/mL. Subsequently, 5 μL of Annexin V-FITC and 5 μL of PI staining solution (from the Annexin V-FITC/PI Apoptosis Detection Kit, Epizyme, China) were added to the cell suspension. The resulting mixture was incubated for 15 min at room temperature under light-protected conditions, after which flow cytometry analysis was performed.

### Enzyme-linked immunosorbent assay

Following the treatment period, supernatants from the cell cultures were harvested and clarified by centrifugation at 1000 rpm for 10 min. Commercial ELISA kits (Elabscience, China) were used to measure interleukin 6 (IL-6) and Tumor Necrosis Factor-α (TNF-α) levels, according to the manufacturer’s instructions. In brief, standards and samples were added to pre-coated plates and incubated at 37 °C for the specified time. Subsequently, the detection antibody, enzyme conjugate, and substrate solution were sequentially added, and the reaction was stopped by adding the stop solution. Absorbance was measured at 450 nm using a microplate reader.

### Determination of oxidative stress indicators

Cellular malondialdehyde (MDA) content and superoxide dismutase (SOD) activity were detected using the corresponding commercial kits (Elabscience, China) according to the manufacturer’s instructions.

### Bioinformatics analysis

Disease-associated genes were obtained from the Gene Cards database (because all scores werehigh). SDG target genes were retrieved from Swiss Target Prediction (probability, > 0.1). The union of disease-associated genes and SDG target genes were intersected via Venn diagram analysis to obtain overlapping candidate target genes. A protein-protein interaction (PPI) network was generated using the STRING platform (confidence score threshold ≥ 0.7) and subsequently visualized using Cytoscape. Topological parameters were calculated as follows: degree, betweenness centrality, and closeness centrality. For functional annotation, the DAVID database was used to carry out Gene Ontology (GO) enrichment and Kyoto Encyclopedia of Genes and Genomes (KEGG) pathway analyses, thereby revealing the biological roles and signaling routes of the candidate target genes. The complete GO and KEGG analyses are shown in **Supplementary Tables 1**-**4**.

### Molecular docking analysis

To verify the direct interaction between SDG and ERBB2, a molecular docking analysis was performed. The three-dimensional structure of ERBB2 was obtained from the Protein Data Bank (PDB) and the structure of SDG was obtained from the PubChem database. The protein model was prepared by removing water molecules and adding hydrogen atoms, and the ligand conformation was optimized using the Gaussian software. Molecular docking was carried out using the Auto Dock Vina software, and the binding affinity between SDG and ERBB2 was evaluated. PyMOL software was used to show the docking posture, and how SDG adapts to the active pocket of ERBB2 was analyzed.

### Cellular thermal shift assay

A CETSA experiment was used to verify the direct combination of SDG and ERBB2 in living cells. First, chondrogenesis was induced; then, ATDC5 cells were treated with 10 μM SDG for 24 h, whereas the control group was treated with the same volume of solvent. The cells were collected, resuspended in PBS, and split into equal parts. Each sample was heated at a temperature gradient of 40-65 °C for 3 min, immediately cooled on ice for 5 minutes after heating, and then the cells were lysed by ultrasound. The supernatant was obtained by centrifugation and the abundance of ERBB2 in the supernatant was evaluated by western blotting. Finally, the thermal stability of ERBB2 was evaluated based on the signal intensity obtained by western blotting.

### Toluidine blue staining

For toluidine blue staining, ATDC5 cells were fixed with 4% paraformaldehyde for 15 min at room temperature. After washing with PBS, cells were stained with 0.1% toluidine blue solution (prepared in 0.1 M acetate buffer, pH 2.5) (Beyotime, China) for 30 min at room temperature. The stained cells were then rinsed with distilled water to remove excess dye and air-dried. Images were captured using an inverted light microscope (Olympus).

### Quantitative real-time polymerase chain reaction

Cellular RNA was isolated using a commercial kit (Biomed, China) and converted to complementary DNA (cDNA) using a reverse transcription system (Biomed, China). Quantitative real-time PCR was carried out using SYBR Green PCR Master Mix (Biomed, China) on a real-time PCR instrument. GAPDH served as the endogenous control (forward 5′- GCCTCCTCCAATTCAACCCT-3,’ reverse 5′- CTCGTGGTTCACACCCATCA-3′), the primer set for ERBB2 was forward 5′-CCCCAGAATACTCCGTTGGAC-3,’ reverse 5′- CCTAGTGGGTGGGGTTTTCC-3′, MMP13 forward 5′-CTTCTTTCTTGTTGAGCTGGACTC-3,’ reverse 5′- CTGTGGAGGTCACTGTAGACT-3. ‘ The 2^-ΔΔCt^ method was used to determine normalized ERBB2 transcript levels.

### Western blot

To prepare the lysates, cells were homogenized in RIPA lysis buffer (GBCBIO, China) and kept on ice for 1 h, followed by centrifugation at 12,000 rpm for 20 min at 4 °C. The resulting supernatants were collected. After quantification, equal amounts of protein (50 μg per lane) were resolved by SDS-PAGE (GBCBIO, China) and electrotransferred onto PVDF membranes (Yeasen, China). Prior to antibody incubation, membranes were treated with blocking buffer for 1 h at room temperature. They were then probed overnight at 4 °C with the primary antibodies (Abcam) and subsequently incubated for 1 hour at room temperature with HRP-conjugated secondary antibodies (Abcam). Immunoreactive signals were developed using an ECL substrate (GBCBIO, China) and captured using a Tanon 5200 chemiluminescence system (Tanon, China). Densitometric analysis of the bands was performed using the ImageJ software, and β-actin was used as the loading control.

### Statistical analysis

Statistical analyses were performed using GraphPad Prism 8.0. The measurement data are expressed as the mean ± standard deviation. Differences between multiple groups were analyzed using one-way analysis of variance (ANOVA) followed by Tukey’s multiple comparisons *post-hoc* test to correct for multiple group comparisons. Differences with *P* < 0.05 were considered statistically significant at p < 0.05. All *in vitro* experiments were independently repeated at least three times.

## Results

### SDG rescues cell viability and collagen II expression in LPS-treated chondrocytes

**Figure 1:**
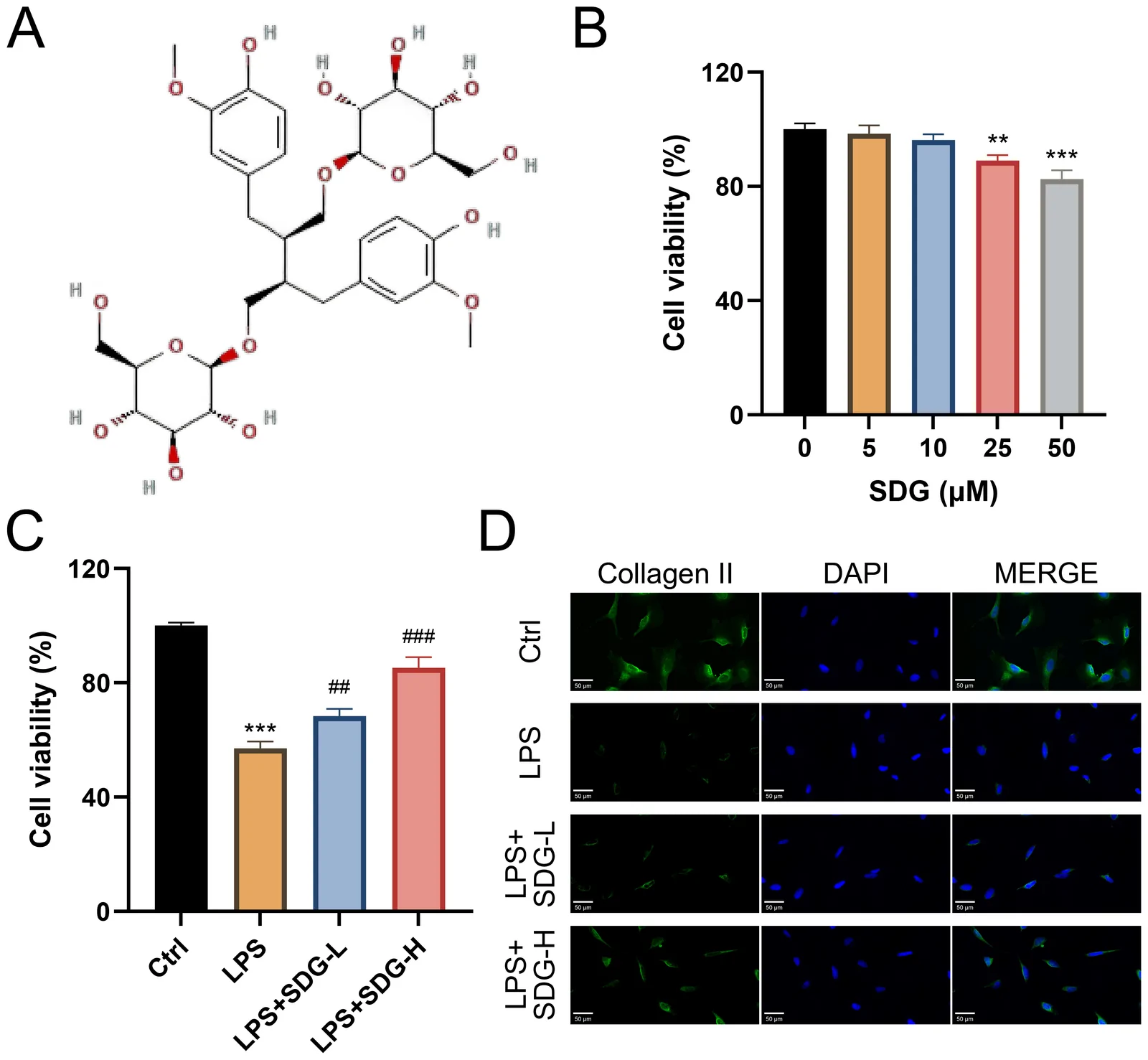
SDG protects ITS induced-ATDC5 cells against LPS-induced injury and promotes chondrogenesis. **(A)** Chemical structure of SDG. **(B)** ITS induced ATDC5 cells were treated with increasing concentrations of SDG (0, 5, 10, 25, 50 μM) for 24 h. Cell viability was measured by CCK-8 assay. ^**^P < 0.01, ^***^P < 0.001 *vs* SDG (0) group. **(C, D)** ITS induced ATDC5 cells were exposed to LPS, followed by treatment with low (5 μM) or high (10 μM) doses of SDG. **(C)** Cell viability assessed by CCK-8 assay. ^***^P < 0.001 *vs* Ctrl group; ^##^*P* < 0.01, ^###^*P* < 0.001 *vs* LPS group. **(D)** Immunofluorescence staining for Collagen II (representative images). Data are presented as mean ± SD (n=3).

**Figure 1A** presents the chemical structure of the SDG. To evaluate the biological activity of SDG, its cytotoxicity and protective efficacy in chondrogenically differentiated ATDC5 cells were assessed. CCK-8 assays showed that SDG had no notable toxicity when applied at doses of ≤10 μM for 24 h, whereas higher concentrations significantly reduced viability. Based on these results, two doses (5 μM and 10 μM) were chosen for further experiments. In LPS-challenged chondrocytes, cell viability was restored by SDG treatment in a dose-dependent manner (**Figure 1C**). LPS significantly reduced collagen II expression, whereas SDG treatment markedly increased collagen II expression, indicating enhanced chondrogenic differentiation, as confirmed by immunofluorescence (**Figure 1D**).

### SDG protects against LPS-induced chondrocyte apoptosis, inflammation and oxidative stress

**Figure 2:**
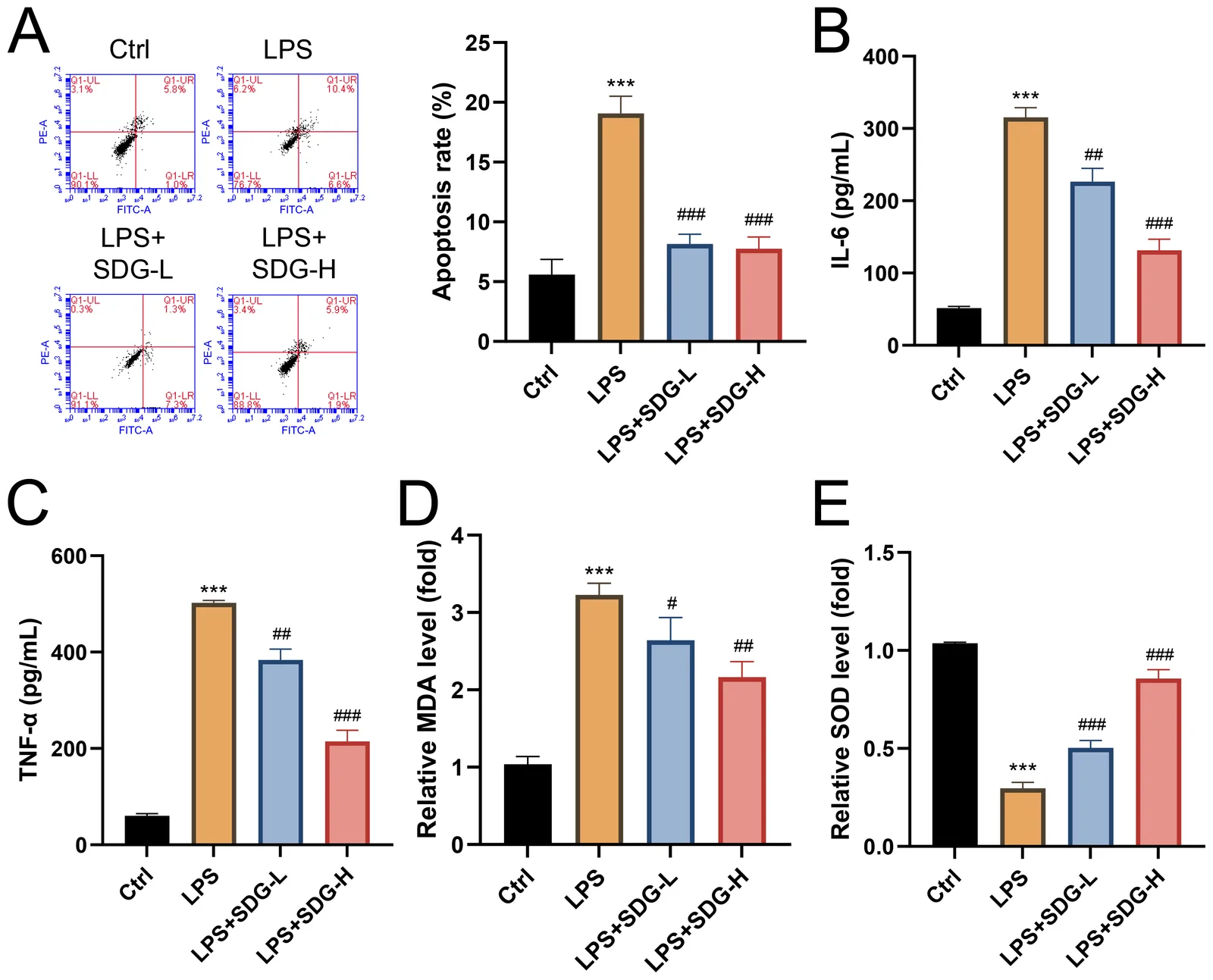
SDG attenuates LPS-induced apoptosis, inflammation and oxidative stress in ITS induced-ATDC5 cells. **(A)** Apoptosis was detected by flow cytometry using Annexin V-FITC/PI double staining. **(B, C)** Levels of pro-inflammatory cytokines IL-6 **(B)** and TNF-α **(C)** in cell culture supernatants were measured by ELISA. **(D, E)** The levels of oxidative stress markers MDA **(D)** and SOD **(E)** were determined using commercial kits. Data are presented as mean ± SD (n=3). ^***^P < 0.001 *vs* Ctrl group; ^#^*P* < 0.05, ^##^*P* < 0.01, ^###^*P* < 0.001 *vs* LPS group.

Next, we examined how SDG influences LPS-triggered apoptosis, inflammation, and oxidative stress. The apoptotic rate of chondrocytes was markedly increased by LPS exposure and this increase was notably reduced by SDG in a concentration-dependent manner (**Figure 2A**). The increased release of IL-6 and TNF-α in LPS-stimulated cell culture supernatants was dose-dependently reduced by SDG (**Figures 2B, C**). Additionally, following SDG treatment, MDA levels decreased markedly, while SOD activity increased, indicating antioxidant activity against LPS-induced oxidative stress (**Figures 2D, E**). Collectively, these results confirm that chondrogenic ATDC5 cells are protected from LPS-induced injury by SDG through the promotion of cell viability and chondrogenesis and the suppression of apoptosis, inflammation, and oxidative stress.

### Bioinformatics analysis and CETSA identify ERBB2 as a potential target of SDG

**Figure 3:**
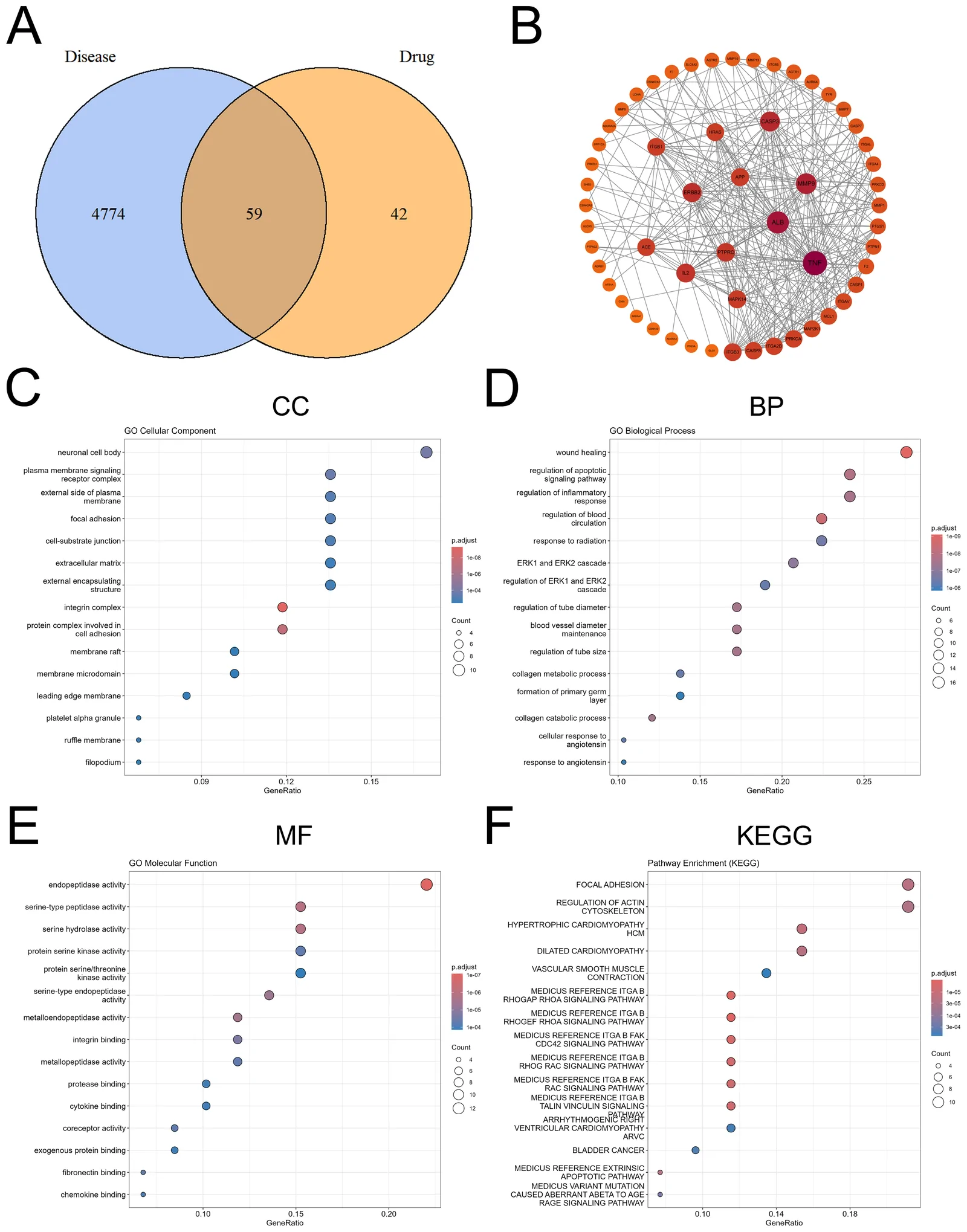
Identification of candidate target genes of SDG and functional enrichment analysis. **(A)** Venn diagram of disease-related genes and SDG target genes. **(B)** PPI network of candidate targets. **(C)** Cellular Component (CC) enrichment analysis. **(D)** Biological Process (BP) enrichment analysis. **(E)** Molecular Function (MF) enrichment analysis. **(F)** KEGG pathway analysis.

To prioritize disease-relevant candidate targets potentially involved in the protective effects of SDG, a series of bioinformatic analyses were performed. First, the predicted SDG targets were intersected with OA-related genes to generate a set of disease-associated candidate targets, resulting in 59 overlapping genes (**Figure 3A**); the specific names of the genes are shown in **Supplementary Table 5**. A PPI network was constructed to analyze the interactions among the candidate targets. Network topology analysis was subsequently performed to evaluate the relative importance of individual nodes. Among these candidates, ERBB2 exhibited a degree value of 23 (ranked fifth), a betweenness centrality of 0.30 (ranked fifth), and a closeness centrality of 0.71 (ranked fifth). ERBB2 was the only gene consistently ranked among the top five across all three topological parameters, suggesting that ERBB2 represents a highly connected candidate node within the PPI network (**Figure 3B**). The results of the complete PPI topology analysis are presented in **Supplementary Table 6**. The specific correlation values for the other genes are shown in **Supplementary Table 1**. Subsequently, GO and KEGG pathway enrichment analyses were performed on candidate target genes (**Figures 3C–F**). GO analysis demonstrated that these candidate genes were markedly enriched in diverse biological processes, predominantly wound healing, regulation of inflammatory responses, and angiogenesis-related vascular remodeling. In terms of cellular components, the enriched terms were primarily concentrated in the plasma membrane, FA, extracellular matrix, and cell junction structures. Candidate target genes were highly correlated with endopeptidase activity, serine peptidase activity, protein kinase activity, and integrin binding function. Further KEGG pathway enrichment results demonstrated that FA was the most significantly enriched signaling pathway. Visualization of the focal adhesion pathway indicated that ERBB2 was involved in this signaling network (**Supplementary Figures 1**, **2**). Furthermore, previous studies have demonstrated that ERBB2 can regulate JAK2/STAT3 signaling, suggesting a potential mechanistic connection between ERBB2-associated focal adhesion signaling and the JAK2/STAT3 pathway (15). To explore the potential therapeutic targets of SDG in osteoarthritis, a compound-target network was constructed based on candidate targets of SDG and OA-related genes. As shown in **Supplementary Figure 3**, SDG was associated with 59 overlapping targets associated with osteoarthritis, indicating that SDG may exert its anti-osteoarthritis effects through a multi-target regulatory mechanism, with ERBB2 identified as one of the candidate targets of interest.

**Figure 4:**
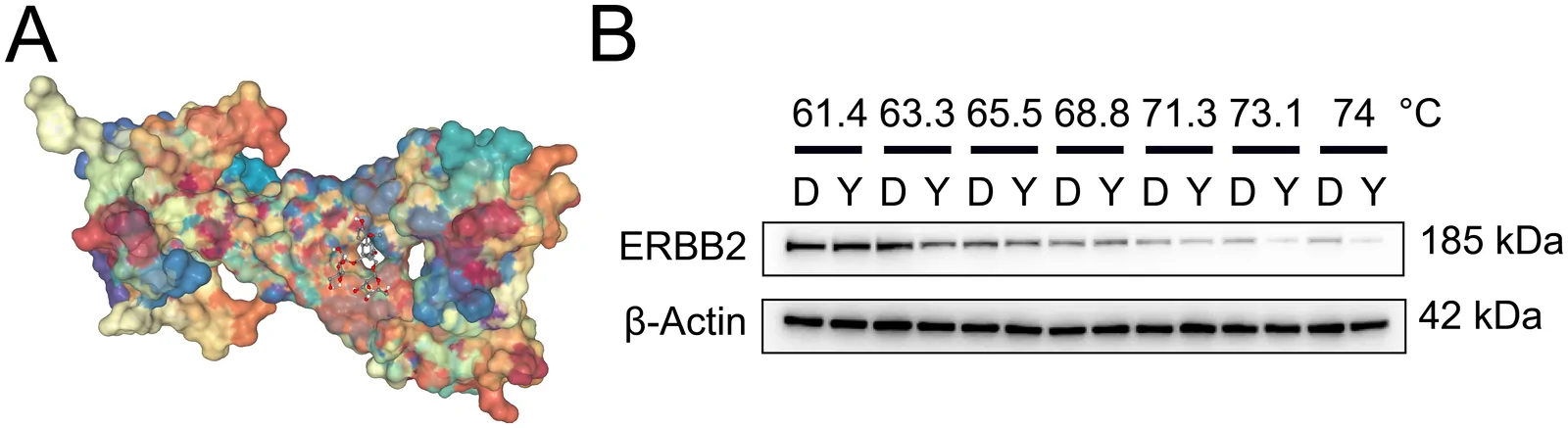
Identification of focal adhesion pathway and direct interaction of SDG with ERBB2. **(A)** Molecular docking of SDG with ERBB2. **(B)** CETSA showing the interaction between SDG and ERBB2. Data are presented as mean ± SD (n=3).

To further investigate the potential interaction between SDG and ERBB2, a molecular docking analysis was performed. The binding energies of the 12 genes with the highest degree of SDG are listed in **Table 1**. Among them, the predicted binding affinity of SDG to ERBB2 was relatively strong, with a Vina score of -8.8 kcal/mol. The results predicted a favorable binding mode between SDG and ERBB2, with a high binding affinity. Subsequently, CETSA was conducted to evaluate the target engagement of ERBB2 by SDG in a cellular context. As shown in **Figure 4B**, SDG treatment markedly increased the thermal stability of ERBB2 compared with that of the control group. These findings support the possibility that SDG interacts with ERBB2 and engages the protein in the cells, although additional biophysical studies are required to definitively establish direct binding.

**Table 1:** Details of molecular docking.

Target gene	Binding energy (kcal/mol)	Exact binding domains	Functional domain	RMSD (Å)	Key amino acid residues	Interaction type
ACE	-9.4	Chain A: 146–173, 276–302, 352–357, 369–387, 411–418, 449–460, 511–530	Peptidase M2 domain (Peptidase_M2, residues 37-625)	0.132	LYS-454, GLU-376	Hydrogen Bonds, Water Bridges
ALB	-8.8	Chain A: 103-104, 106-108, 148, 150, 153, 157, 191-193, 195-201, 204-206, 211, 214, 218-219, 221-223, 238, 242, 257, 260, 264, 287-295, 339-341, 343-344, 436-437, 440, 444-445, 447-452, 454-455, 461-466, 477-478	Serum albumin family domain (Albumin, residues 19-609)	2.679	Tyr-150, His-242, Arg-257, Lys-195, Lys-199, Arg-218, Arg-222; Arg-410, Ser-489, Lys-414	—
APP	-6.8	Chain B: 31,116,118-119,123-128,167 Chain A: 81,83-84,119-122,172-176,178,180	Multi-domain protein: E1 domain (residues 18-190), E2 domain (residues 295-500)	15.368	Cys-144	
CASP3	-5.9	Chain A: 101-102,104-109,141-144,146-151	Caspase family p20 domain (Caspase_p20, residues 54-178)	0.088	Cys-163, His-121	Hydrophobic Interactions
ERBB2	-8.8	Chain B: 725-734,751-753,774,783-785,796-805,807-808,845,849-850,852,862-866,885,1003-1004	Tyrosine kinase domain (TKD, residues 720-976)	0.874	Lys-753, Met-801, Asp-863	Hydrophobic Interactions, Hydrogen Bonds, Water Bridges, Halogen Bounds
HRAS	-9.3	Chain A: 10-18,21,28-35,60,83,85-86,89,116-120,144-148	Ras family G-domain (Ras, residues 5-165)	0.363	Gly-12, Gly-13, Gln-61	Metal Complexes
IL2	-5.4	Chain A: 19,22-23,25-27,29-32,34-35,38,71-74,77-84	Interleukin-2 family domain (IL-2, residues 1-133)	0.457	Lys-35, Arg-38	Hydrogen Bonds, Salt Bridges
ITGB1	-7.9	Chain B: 56-60,82-84,91-92,139,141,451-453,455,479-482 Chain A: 192,292-295,310,312-314	Integrin beta chain VWA domain (VWA, residues 34-377)	4.065	Asp-139, Ser-124	—
MAPK14	-6.7	Chain A: 5-8,21-24,45,87-95,342,345-350	Protein kinase domain (Pkinase, residues 24-308)	0.834	Thr-180, Tyr-182	Hydrophobic Interactions,Hydrogen Bonds,Water Bridges, π-Cation Interactions,Halogen Bonds
MMP9	-7.8	Chain B: 47,50-52,100-115,175,177-181,187,190-194,229-236,266-267	Catalytic domain (Phe107-Pro449), Zn-binding site	0.283	His-401, His-405, His-411, Glu-402	Metal Complexes
PTPRC	-8.5	Chain B:764-767,801-805,808,894,929,932,957-958,997-998,1000-1006,1159-1160,1163,1167,1169,1171,1174-1182	Tyrosine-protein phosphatase domain (Y_phosphatase, residues 675-909)	0.790	Cys-828, Arg-830	—
TNF	-8.1	Chain A: 69,75,97-102,114-116 Chain B: 69,98-101,114-116 Chain C: 68-69,98-102,111-116 Chain H: 103	TNF homology domain (THD, residues 88-243)	13.834	Tyr-119, Gln-127	Hydrophobic Interactions,Hydrogen Bonds,Water Bridges, π-Cation Interactions,Halogen Bonds

### SDG suppresses the ERBB2/JAK2/STAT3 signaling pathway in LPS-treated chondrocytes

**Figure 5:**
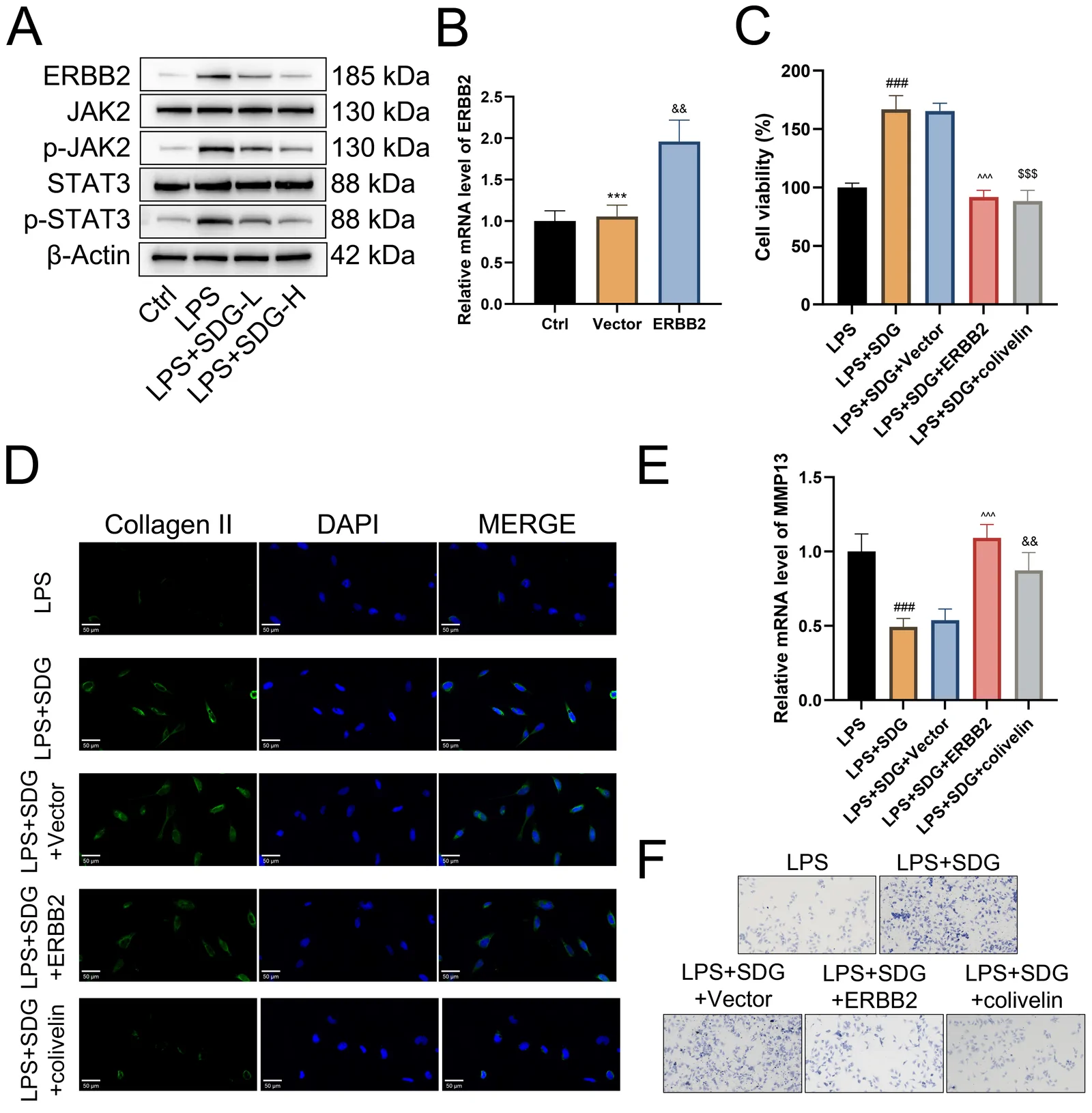
SDG binds to ERBB2 and regulates cell viability and matrix production via ERBB2/JAK3/STAT3 signaling. **(A)** Western blot was used to detect the protein levels of ERBB2, p-JAK2, JAK2, p-STAT3, STAT3, and β-actin. **(B)** qRT-PCR validation of ERBB2 overexpression efficiency. ^***^P < 0.001 *vs* Ctrl group; ^&&^*P* < 0.01 *vs* Vector group. **(C, D)** ITS-induced ATDC5 cells were transfected with NC or ERBB2 overexpression plasmid, then exposed to LPS and treated with SDG (10 μM) in the presence or absence of colivelin (0.5 μM, a JAK/STAT activator). **(C)** Cell viability measured by CCK-8 assay. **(D)** Immunofluorescence staining for Collagen II (representative images). **(E)** The expression of MMP13 was determined by qRT-PCR. **(F)** Toluidine blue staining of sulfated proteoglycans in ATDC5 chondrocyte. Data are presented as mean ± SD (n=3). ^###^*P* < 0.001 *vs* LPS group; ^^^^^*P* < 0.001 *vs* LPS + SDG + Vector group; ^$$$^*P* < 0.001 *vs* LPS + SDG group.

To determine whether the regulatory effect of SDG on the JAK2/STAT3 pathway is mediated by ERBB2, we first examined the functional consequences of ERBB2 knockdown in the absence of SDG treatment. As shown in **Supplementary Figure 4A**, siRNA-mediated knockdown of ERBB2 was confirmed using qRT-PCR. Under LPS-stimulated conditions, ERBB2 silencing resulted in a marked reduction in JAK2 and STAT3 phosphorylation (**Supplementary Figure 4B**), whereas total JAK2 and STAT3 protein levels remained unchanged. Furthermore, ERBB2 knockdown significantly decreased IL-6 secretion in the culture supernatant compared with that in the LPS control group (**Supplementary Figure 5**). These findings indicated that ERBB2 functions as an upstream regulator of the JAK2/STAT3 signaling axis and IL-6 production in LPS-stimulated ATDC5 chondrocytes. To further determine whether SDG recapitulates these effects, we examined the effect of SDG treatment on this pathway. As shown in **Figure 5A**, SDG treatment reduced ERBB2 protein levels in a dose-dependent manner and suppressed the phosphorylation of both JAK2 and STAT3, whereas total JAK2 and STAT3 remained unchanged. These results suggest that SDG may exert its anti-inflammatory effects, at least in part, through inhibition of the ERBB2/JAK2/STAT3 signaling pathway.

### SDG exerts chondroprotective effects via the ERBB2/JAK2/STAT3 signaling axis

**Figure 6:**
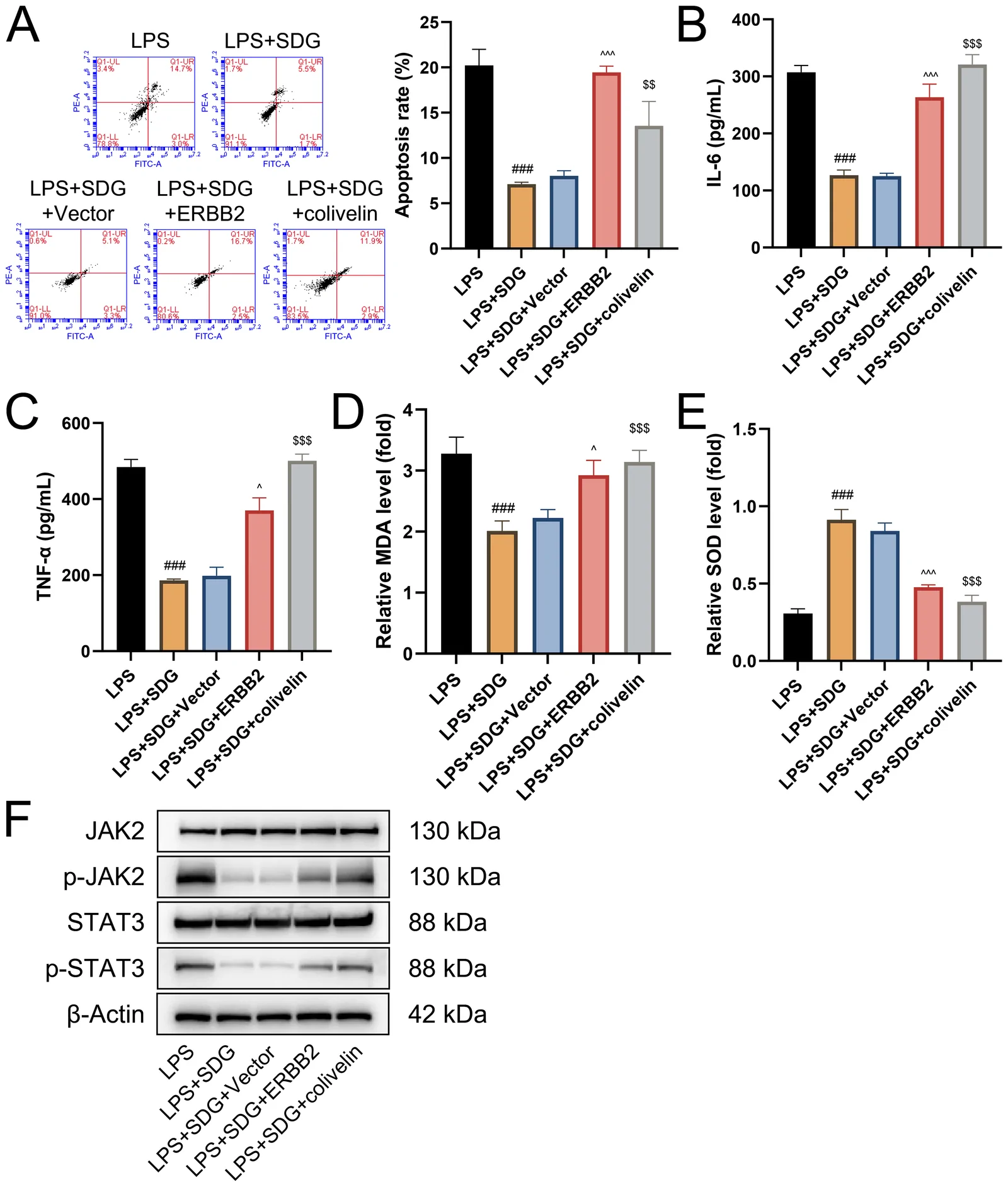
SDG suppresses apoptosis, inflammation and oxidative stress via the ERBB2/JAK2/STAT3 axis in LPS-treated chondrogenic differentiated ATDC5 cells. ITS-induced ATDC5 cells were transfected with NC or ERBB2 overexpression plasmid, then exposed to LPS and treated with SDG (10 μM) in the presence or absence of colivelin (0.5 μM, a JAK/STAT activator). **(A)** Apoptosis was detected by flow cytometry using Annexin V-FITC/PI double staining. **(B, C)** Levels of pro-inflammatory cytokines IL-6 **(B)** and TNF-α **(C)** in cell culture supernatants were measured by ELISA. **(D, E)** The levels of oxidative stress markers MDA **(D)** and SOD **(E)** were determined using commercial kits. **(F)** Western blot was used to detect the protein levels of p-JAK2, JAK2, p-STAT3, STAT3, and β-actin (loading control). ^###^*P* < 0.001 *vs* LPS group; ^^^*P* < 0.05, ^^^^^*P* < 0.001 *vs* LPS + SDG + Vector group; ^$$^*P* < 0.01, ^$$$^*P* < 0.001 *vs* LPS + SDG group. Data are presented as mean ± SD (n=3).

To determine whether the ERBB2/JAK2/STAT3 signaling axis was involved in mediating the protective effects of SDG, rescue experiments were performed. First, an ERBB2 overexpression plasmid was constructed and its overexpression efficiency was verified by qRT-PCR (**Figure 5B**). ATDC5 cells received either NC or ERBB2 plasmid overexpression, followed by LPS exposure and SDG (10 μM) treatment in the presence or absence of colivelin (0.5 μM, a JAK/STAT activator). According to CCK-8 measurements, the viability-preserving effect of SDG against the LPS challenge was partially reversed by ERBB2 overexpression or colivelin administration (**Figure 5C**). The promotive effect of SDG on Collagen II expression was reversed by ERBB2 overexpression or colivelin treatment, as shown by immunofluorescence staining (**Figure 5D**). qRT-PCR analysis demonstrated that SDG significantly reduced MMP13 mRNA levels, whereas cotreatment with oe-ERBB2 or colivelin markedly reversed this suppression (**Figure 5E**). Toluidine blue staining further confirmed these findings; SDG enhanced extracellular matrix proteoglycan deposition (increased staining intensity), which was diminished by oe-ERBB2 or colivelin (**Figure 5F**). Flow cytometry analysis showed that the inhibitory effect of SDG on LPS-induced apoptosis was significantly reversed by ERBB2 overexpression or colivelin treatment (**Figure 6A**). ELISA results revealed that the suppressive effect of SDG on the secretion of pro-inflammatory cytokines IL-6 and TNF-α was abrogated by ERBB2 overexpression or colivelin treatment (**Figures 6B, C**). Additionally, the antioxidant effect of SDG was reversed by ERBB2 overexpression or colivelin treatment, as evidenced by increased MDA levels and decreased SOD activity (**Figures 6D, E**). Western blot analysis confirmed that JAK2/STAT3 phosphorylation was inhibited by SDG and was significantly upregulated by ERBB2 overexpression or colivelin treatment (**Figure 6F**).

To further elucidate the mechanistic link between ERBB2 and JAK2/STAT3 activation, we examined whether ERBB2 overexpression-induced JAK2/STAT3 phosphorylation was mediated through the IL-6/IL-6R axis. To this end, LPS-stimulated ATDC5 cells were transfected with oe-ERBB2 in the presence or absence of tocilizumab (an IL-6R blocking antibody), and JAK2/STAT3 phosphorylation was assessed using western blotting. As shown in **Supplementary Figure 6**, ERBB2 overexpression alone significantly increased JAK2 and STAT3 phosphorylation, whereas co-treatment with tocilizumab largely reversed this effect without affecting total JAK2 or STAT3 levels. These results suggest that ERBB2 activates JAK2/STAT3 signaling, at least in part, through the IL-6/IL-6R axis. Taken together, these findings indicate that SDG exerts chondroprotective effects, at least partially, through modulation of ERBB2 and its downstream JAK2/STAT3 signaling pathway. These changes are associated with the attenuation of apoptosis, inflammatory responses, and oxidative stress, as well as the preservation of the chondrogenic phenotype.

## Discussion

Naturally occurring polyphenolic compounds have attracted growing interest as agents for preventing non-communicable chronic diseases (NCDs), largely because of their capacity to counter oxidative stress and modulate inflammation (22). However, a major challenge in translating these beneficial effects into clinical practice is that the specific molecular targets of most polyphenolic compounds remain unclear. Lignans, a subclass of these compounds, have shown promise in mitigating various NCDs, including OA (23, 24). It is worth noting that natural SDG, as a diglycoside lignan, exhibits limited cell membrane permeability. In contrast, its hydrolysis aglycone ring-opening isolariciresinol (SECO) easily passes through the cell membrane through passive diffusion and interacts with its potential target molecules (25, 26). This difference in membrane permeability is of great significance for explaining the intracellular targets of SDG-related biological activity because the cellular uptake of aglycone forms may be a prerequisite for binding to intracellular proteins such as ERBB2. In this study, SDG alleviated LPS-induced inflammatory injury in chondrocytes and was associated with reduced ERBB2 expression and suppression of JAK2/STAT3 signaling. These findings provide preliminary evidence supporting the involvement of ERBB2-associated signaling in the cellular actions of SDG.

Our study demonstrated that SDG significantly restored cell viability and promoted collagen II expression in LPS-challenged chondrocytes in a concentration-dependent manner, while dose-dependently inhibiting apoptosis, decreasing the release of pro-inflammatory cytokines, and attenuating oxidative stress. These findings closely align with those of previous studies on the bone-protective effects of SDG. For instance, Chen et al. found that SDG ameliorated ovariectomy-induced osteoporosis in rats by regulating estrogen receptor (ERα and ERβ) expression, reducing bone injury and inflammation, and improving bone formation indexes (27). In addition, SDG was reported to significantly inhibit IL-1β induced inflammatory factor expression, promote collagen II expression, and suppress MMP13 and ADAMTS5 expression and the NF-κB pathway, thereby alleviating cartilage degradation (8). Collectively, these studies indicate that SDG exerts beneficial chondroprotective effects against OA.

The present study suggests that ERBB2 may serve as an important molecular target for the chondroprotective effects of SDG. Our data showed that SDG was associated with ERBB2 engagement and reduced ERBB2 protein expression, accompanied by attenuation of JAK2/STAT3 signaling activation. Given the emerging evidence implicating ERBB2 in OA pathogenesis, these findings provide a potential mechanistic link between SDG treatment and modulation of inflammatory responses in chondrocytes. Recent bioinformatics and experimental evidence has identified ERBB2 as a key regulator of OA progression. For instance, through multi-omics analysis, Cai et al. found that ERBB2 is upregulated in OA cartilage, its expression is positively correlated with OA severity, and possesses a high diagnostic value (28). A study by Rana et al. further revealed the pathogenic involvement of ERBB2 in OA, showing that downregulation of miR-4505 and miR-331-3p leads to upregulation of their common target ERBB2, thereby exacerbating catabolic and inflammatory processes in OA (13). Additionally, Wang et al. pointed out through bioinformatics analysis that ERBB2 plays a critical role in OA progression in postmenopausal women and, owing to its strong association with the disease, is regarded as a promising therapeutic target (29). The study by Hallbeck et al. provides another line of evidence supporting the pathogenicity of ERBB2, demonstrating that overexpression of ERBB2 in joint tissues induces aberrant growth patterns, leading to inflammatory synovial hyperplasia and aggravated joint destruction (30). Collectively, these studies suggest that ERBB2 is closely associated with OA progression, and may represent a promising therapeutic target for disease intervention.

We further found that the inhibition of ERBB2 by SDG markedly reduced p-JAK2 and p-STAT3 levels. Moreover, the protective actions of SDG were counteracted by either ERBB2 overexpression or colivelin, a known activator of the JAK/STAT axis. These results confirmed that the ERBB2/JAK2/STAT3 axis is the core signaling pathway mediating the action of SDG. The JAK/STAT pathway is widely recognized as a key driver of inflammation and apoptosis in OA (14, 31, 32), and ERBB2 activates JAK/STAT signaling (15, 33). Notably, SDG exhibits inflammation-suppressing properties via JAK/STAT modulation in various pathological contexts. For instance, Yu et al. demonstrated that SDG, through its gut bacterial metabolite enterolactone, suppressed Th2 immune responses in atopic dermatitis via suppression of the JAK2-STAT6 signaling cascade (34). Moreover, the present study further revealed that the antioxidant effects of SDG, as well as its promotion of collagen II expression and restoration of chondrocyte viability, are dependent on this signaling axis. This is consistent with previous reports that the JAK/STAT pathway is involved in regulating oxidative stress, matrix synthesis, and cell survival in chondrocytes (14, 35, 36). In summary, the ERBB2/JAK2/STAT3 axis is indispensable for SDG’s protective effects of SDG against LPS-stimulated chondrocyte injury, encompassing the suppression of apoptosis, inflammation, and oxidative stress as well as the promotion of collagen II expression.

Although the present study yields important insights, several constraints should be considered. First, the experiments were performed using an *in vitro* cell model. ATDC5 cells acquire a chondrocyte-like phenotype following ITS induction; nevertheless, they do not fully replicate primary human chondrocytes or the native joint environment *in vivo*. Accordingly, additional animal studies are needed to confirm the protective efficacy and pharmacokinetic profile of SDG in animal models of OA. Second, it should be noted that the overlap between the predicted SDGs and osteoarthritis-related genes is used as a candidate priority strategy rather than a clear target recognition method. Since direct ligand-binding targets do not necessarily show altered expression in OA, this workflow may miss some SDG targets related to biology. CETSA primarily measures ligand-associated thermal stabilization of proteins and cannot independently distinguish between direct binding and indirect stabilization mechanisms. Therefore, although the combined molecular docking and CETSA results support ERBB2 target engagement by SDG, further biophysical assays such as surface plasmon resonance (SPR), microscale thermophoresis (MST), and isothermal titration calorimetry (ITC) are necessary to conclusively characterize the binding interaction.

In summary, the present study provides preliminary evidence that SDG attenuates LPS-induced inflammatory injury in chondrocytes through modulation of ERBB2 and the downstream JAK2/STAT3 signaling pathway. Mechanistically, molecular docking and CETSA analyses supported the potential interaction between SDG and ERBB2, whereas SDG treatment was associated with reduced ERBB2 expression and decreased activation of JAK2/STAT3 signaling, thereby alleviating inflammation, oxidative stress, apoptosis, and extracellular matrix degradation. These findings expand our understanding of the molecular actions of SDG and suggest that ERBB2-associated JAK2/STAT3 signaling may contribute to its chondroprotective effects under inflammatory conditions. Nevertheless, the current study was based on an LPS-induced inflammatory cell model, which primarily reflects acute inflammatory responses, rather than the complex pathophysiology of chronic OA. Therefore, further investigations using mechanically induced, aging-related, or surgically induced OA models, as well as *in vivo* studies, are required to determine the therapeutic relevance of SDG in chronic OA progression.

## Data Availability

The original contributions presented in the study are included in the article/**Supplementary Material**, further inquiries can be directed to the corresponding author/s.

## Supplementary material

Supplementary Figure 1 Focal adhesion signaling pathway (Source: KEGG database, hsa04510).

Supplementary Figure 2 The expression and mapping of key genes in the Focal adhesion signaling pathway. The red highlight in the figure represents a highly expressed gene.

Supplementary Figure 3 Compound-target network of SDG against osteoarthritis.

Supplementary Figure 4 Low expression of ERBB2 regulates the JAK2/STAT3 signaling axis. **(A)** qRT-PCR was used to determine the knockdown efficiency of ERBB2. **(B)** Western blot was used to measure the expression of proteins related to the JAK2/STAT3 signaling axis. ^###^*P* < 0.001 vs si-NC group.

Supplementary Figure 5 The effect of ERBB2 low expression on the IL-6 content in the supernatant of LPS-stimulated ATDC5 chondrogenic cells. ^###^*P* < 0.001 vs si-NC group.

Supplementary Figure 6 Western blot was used to determine the expression of proteins related to the JAK2/STAT3 signaling pathway.

Supplementary Table 1 GO analysis of specific details in BP.

Supplementary Table 2 GO analysis of specific details in CC.

Supplementary Table 3 GO analysis of specific details in MF.

Supplementary Table 4 KEGG analysis of specific details.

Supplementary Table 5 Specific names of 59 overlapping genes.

Supplementary Table 6 PPI network topology analysis results details.
